# Hypo-Osmoregulatory Roles of Vasotocinergic and Isotocinergic Systems in the Intestines of Two European Sea Bass Lineages

**DOI:** 10.3390/ijms232113636

**Published:** 2022-11-07

**Authors:** Quanquan Cao, Eva Blondeau-Bidet, Catherine Lorin-Nebel

**Affiliations:** 1 MARBEC, Univ Montpellier, CNRS, IFREMER, IRD, 34095 Montpellier, France; 2 College of Animal Science and Technology, Sichuan Agricultural University, Chengdu 611130, China

**Keywords:** mRNA, arginine vasotocine receptor, isotocin receptor, intestine, osmoregulation, *Dicentrarchus labrax*, intraspecific differences, neuropeptide receptors

## Abstract

European sea bass (*Dicentrarchus labrax*) are a major aquaculture species that live in habitats with fluctuating salinities that are sometimes higher than in seawater (SW). Atlantic and West-Mediterranean genetic lineages were compared regarding intestinal neuropeptide receptor expression in SW (36%) and following a two-week transfer to hypersalinity (HW, 55%). Phylogenetic analysis revealed seven neuropeptide receptors belonging to the arginine vasotocine (AVTR) family and two isotocin receptors (ITR). Among AVTR paralogs, the highest mRNA levels were recorded for *v1a2*, with a two- to fourfold upregulation in the European sea bass intestinal sections after transfer of fish to HW. Principal component analysis in posterior intestines showed that *v1a2* expression grouped together with the expression and activity of main ion transporters and channels involved in solute-coupled water uptake, indicating a possible role of this receptor in triggering water absorption. *v1a1* expression, however, was decreased or did not change after transfer to hypersaline water. Among ITR paralogs, *itr1* was the most expressed paralog in the intestine and opposite expression patterns were observed following salinity transfer, comparing intestinal sections. Overall, different expression profiles were observed between genetic lineages for several analyzed genes which could contribute to different osmotic stress-related responses in *D. labrax* lineages.

## 1. Introduction

Arginine vasotocine (AVT) (homologue to mammalian vasopressin) and isotocin (IT) (homologue to mammalian oxytocin) neuroendocrine systems are nonapeptides produced in neurons of the hypothalamic preoptic and lateral tuberal nuclei and are stored and released by the neurohypophysis into the circulation [1,2,3]. They are involved in social behaviors, diet regulation, acid-base regulation and salt balance [4,5,6].

Vasotocinergic and isotocinergic systems have been suggested multiple times to be involved in triggering stress-related responses in fish, for example following air exposure [7], starvation [8] and salinity challenges [9,10,11]. Plasma AVT concentrations often correlate positively with plasma osmolality, indicating the importance of this hormone in hyperosmotic environments [12,13,14,15,16]. AVT also plays a role in water balance by altering glomerular filtration rate and urine flow rate [17]. AVT and IT have been shown to regulate water transport via AQP1 in the sea bream intestine [18]. The role of IT in the regulation of internal osmotic and ionic homeostasis, however, remains poorly investigated in fish [14].

The endocrine effects of AVT and IT rely on the presence of membrane receptors [18]. Several AVP receptors have been described in the ancestral gnathostome lineage, comprising four distinct subtypes (V1A, V1B, V2A, V2B) [19,20]. The V1B receptor mediates the stimulatory effects of AVP on adrenocorticotropic hormone (ACTH) release through the pituitary under basal and stress conditions and is therefore a key regulator of the hypothalamic–pituitary–adrenal (HPA) axis in mammals [21]. Mammalian V2 receptor regulates water retention in renal collecting ducts expressing aquaporins [22].

Teleost fishes have multiple nonapeptide receptors, including two V1A-type receptors, up to five V2-type receptors and two IT receptors [16,20,23,24,25,26]. However, the roles of these receptors are not well known in fish. In the marbled eel (*Anguilla marmorata*), *itr* exhibited distinct time-course expression patterns and showed salinity sensitivity in the brain, gills and intestine [27]. NKA, NKCC2 and aquaporins have been mentioned to be functionally linked and regulated by different AVT receptors (AVTR) to facilitate ion and water transport in osmoregulatory tissues such as gills [18], kidneys [11] and intestines [15].

The endocrine control of solute-coupled water transport in the intestine has been investigated in a few species only, mainly the gilthead sea bream [11,15,28]. Studies need to be extended to other marine species. In sea bream, V1A2- and V2 receptors were highly expressed in the intestine [11] with higher mRNA levels of the V2-type receptor in the anterior versus the posterior intestine. An inverse tendency was observed for V1A2, where higher transcript levels were measured in posterior sections (rectum) compared to the middle intestine. In *Xenopus* oocyte swelling assays, sea bream AVT stimulates oocyte swelling through AQP1a or AQP1b in the presence of AVTR V2 [18]. Similarly, sea bream IT has been suggested to regulate AQP1a function [28,29].

*D. labrax* from North Atlantic (A) and West Mediterranean (M) lineages have evolved in habitats with slightly different salinity and temperature regimes and, in the context of climate change, these differences will become even more contrasted as the Mediterranean sea is becoming warmer and saltier [30,31,32,33]. Climate change is one of the main reasons leading to the appearance of hypersaline environments in warm temperate or tropical regions [34]. Hypersalinity is extremely stressful in fish and triggers complex stress-related responses. The effect of hypersalinity on the structure and function of osmoregulatory organs in fish has been addressed by several authors [35]. In our previous study conducted on both *D. labrax* lineages, Na^+^/K^+^-ATPase (NKA) activities and mRNA expressions of *nkaα1*, *nkcc2* and several aquaporins (*aqp8b*, *aqp8aa*, *aqp8ab*, *aqp1a* and *aqp1b*) changed significantly according to hypersalinity acclimation in the posterior intestine [36]. Comparing posterior intestines of North Atlantic (A) and West Mediterranean (M) genetic lineages of *D. labrax*, we mainly observed differences in the expression patterns of these genes in seawater and much less in hypersaline water. This indicates different physiological strategies of *D. labrax* genetic lineages in their native salinity regarding solute-regulated water transport [36].

In this study, we will explore *D. labrax* vasotocinergic and isotocinergic systems by generating a phylogenetic tree from mRNA sequences of arginine vasotocin (AVT) and isotocin (IT) receptors present in *D. labrax* relative to other vertebrates. Specific domains involved in the binding of AVT and IT will be compared with sequences of other species. We will then explore the roles of AVTR and ITR in salinity acclimation at the intestinal level by examining gene expression patterns in *D. labrax* acclimated to seawater (SW, 36%) or hypersaline water (HW, 55%) for two weeks. We will also compare expression patterns of AVTR and ITR in Atlantic (A) and Mediterranean sea bass (M) to investigate intraspecific differences. Finally, our data on vasotocinergic and isotocinergic systems will be linked to previous data obtained from this species regarding solute-coupled water uptake in order to investigate possible roles of neuropeptide hormone receptors expression changes in intestinal water uptake at high salinity. This study aims to compare endocrine control of solute-coupled water uptake between sea bass lineages and their responses following high salinity challenges by focusing on vasotocinergic and isotocinergic systems.

## 2. Results

*D. labrax* analyzed in this and a previous study were characterized by a mean length of 18.30 ± 6.49 g and a mean weight of 27.65 ± 13.51 g [37]. Mortality was low with one death observed in both the MSW and MHW groups after 1 and 4 days of transfer. No signs of pathology were observed.

### 2.1. Phylogenetic Analysis

**Figure 1:**
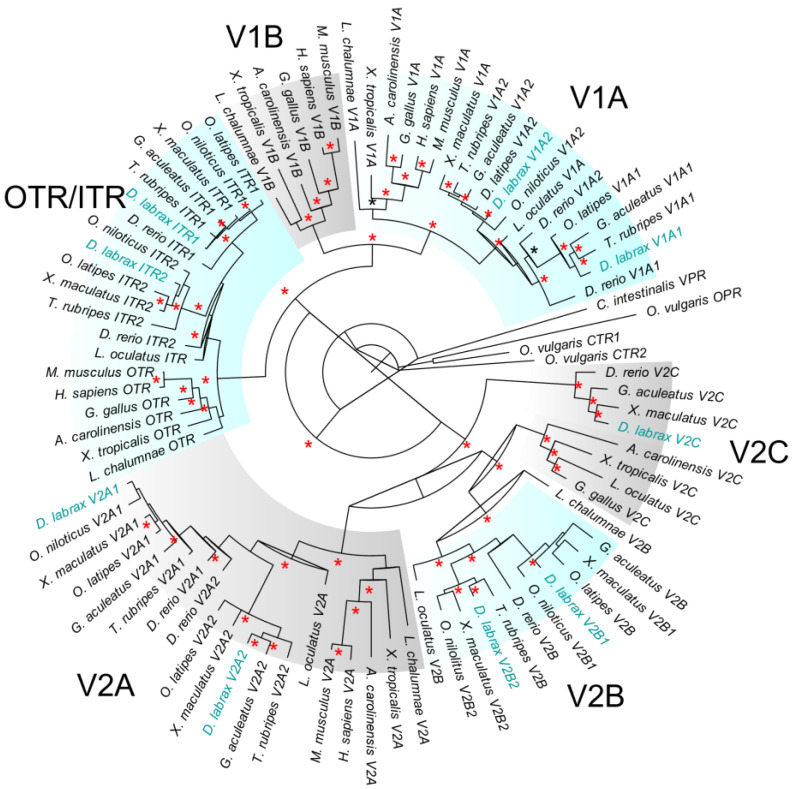
Phylogenetic tree of AVTRV-1A1, -1A2, -2A1, -2A2, -2B1, -2B2, -2C, ITR1 and ITR2. Sequences from European sea bass are indicated in blue. Branch lengths represent the degree of divergence. Bayesian posterior probabilities (bpp) are indicated in nodes as red asterisks (if bpp ≥0.99) and in black asterisks (if 0.95 ≤ bpp ≥ 0.98). Nodes with bpp <0.95 are not annotated.

The phylogenetic tree derived from amino acid sequences shows the relationships of the seven AVT receptors and two IT receptors of *D. labrax* relative to selected nonapeptide receptors from other animals (Figure 1). Phylogenetic relationships illustrate the presence of two V1A-type receptors (V1A1 and V1A2), five V2-type receptors (V2A1, V2A2, V2B1, V2B2 and V2C), and two IT receptors (ITR1 and ITR2) in *D. labrax*. Sixty-one nodes were strongly supported with a Bayesian posterior probability (BPP) greater than 0.90, eight nodes showed medium support (0.79–0.89) and thirteen nodes were not supported (BPP values between 0.73 and 0.50). The phylogenetic analysis revealed four main clades. The first one was composed of three clusters with both V1A-type receptors (V1A1, V1A2) and the mammal V1A-type receptor. The second clade included the two IT receptors in fish and the mammal’s oxytocin receptor (OTR). The third clade was divided into two groups with the V2A1 receptors including the sea bass V2A1 receptor and the V2A2 receptors. The last clade is represented by the V2B1 and V2B2-type receptors. The V2C receptor formed a separate branch.

### 2.2. Amino Acid Analysis in Regions Involved in Ligand Binding

Amino acid sequences of V1A1, V1A2 and ITR1 were compared in *D. labrax* and with other fish and mammalian species (Figure S1). Among OXT/AVP (Figure S1, black asterisks) or AVT (Figure S1, red asterisks) ligand binding amino acids identified and compared in previous studies [37,38,39,40], *D. labrax* V1A1, V1A2 and ITR1 were very conserved in N-terminus/TM1, TM2/ECL1, TM3/ICL2, ECL2, ICL3, TM6/ECL3. TM7 was not analyzed as the *D. labrax* V1A2 sequence was incomplete. We identified 21 amino acids that differ between *D. labrax* V1A1 and V1A2 in those regions indicated by green boxes in Figure S1. Among them, only one amino acid (at position 124, in the Intracellular loop 3 (ICL3)) corresponds to that previously identified as the AVP/OXT binding amino acid. All six important residues identified previously for oxytocin ligand selectivity, binding and signaling are well conserved in *D. labrax* ITR1, indicated by red boxes. These amino acids are also very well conserved in AVTR, except at position 209.

### 2.3. mRNA Expression of avtr and itr Paralogs

**Figure 2:**
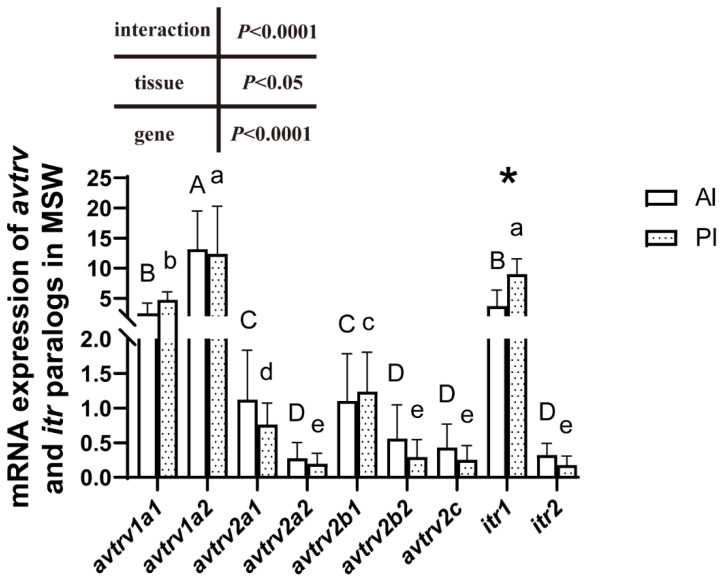
Relative *avtrv* and *itr* mRNA expression in the anterior (AI) and posterior (PI) intestines of Mediterranean European sea bass maintained in seawater. Within the intestinal region, columns displaying different letters are significantly different. Upper letters were indicated for anterior intestines and lower letters were indicated for posterior intestines. Asterisks indicated expression differences between AI and PI for a given gene (two-way ANOVA followed by Tukey’s test, *p* < 0.05, *n* = 11).

Gene-specific distributions of AVTR and ITR were examined in the anterior and posterior intestines of SW-acclimated Mediterranean sea bass (Figure 2). *avtrv1a2* was at least three times more expressed than the other *avtr* in the anterior and posterior intestines. *avtrv1a1* was the second most expressed *avtr* paralog in both intestinal regions whereas the other *avtr* showed at least five times lower expression levels. Among isotocin receptors, *itr1* was around 50 and 80 times more expressed than *itr2*, respectively, in the anterior and posterior intestine. *itr1* was more highly expressed in the posterior than in the anterior intestine. A significant effect of the gene (*p* < 0.0001) and tissue differences (*p* < 0.05) were observed (Figure 2).

### 2.4. avtr and itr mRNA Expression in the Anterior Intestine

**Figure 3:**
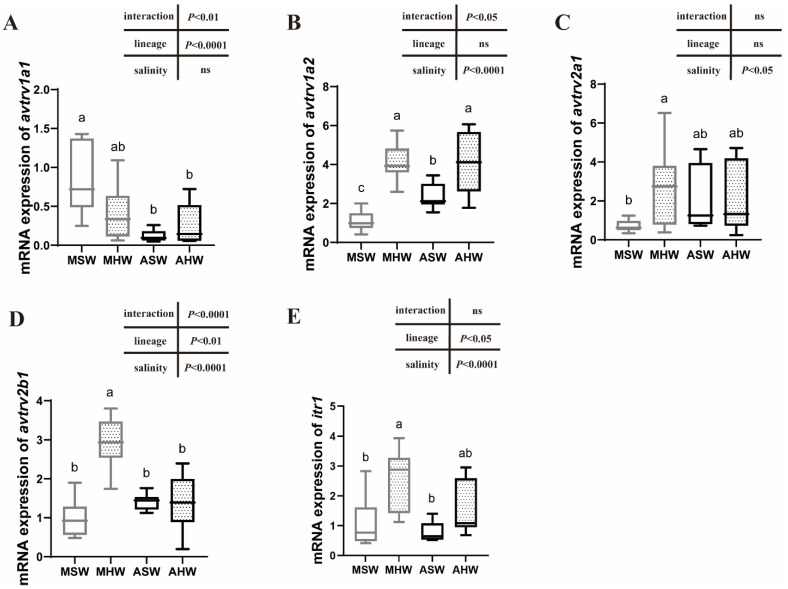
Relative *avtrv1a1* (**A**), *avtrv1a2* (**B**), *avtrv2a1* (**C**), *avtrv2b1* (**D**) and *itr1* (**E**) mRNA expression were measured in the anterior intestines of Mediterranean (M) and Atlantic (A) European sea bass maintained in seawater (SW) and hypersaline water (HW). Different letters denote significant differences between groups (two-way ANOVA followed by Tukey’s test, *p* < 0.05, *n* = 11). Data are represented as the median, first and third quartiles (box), minimum and maximum values. ns: non significant.

In the anterior intestine, MSW showed significantly higher *avtrv1a1* levels than fish from ASW and AHW conditions. A significant lineage effect (*p* < 0.0001) was observed for *avtrv1a1* with higher transcript levels in Mediterranean sea bass than in Atlantic sea bass (Figure 3A). No salinity effect was recorded for this gene. A significantly higher *avtrv1a2* expression was observed in HW compared to SW groups (Figure 3B) (salinity effect: *p* < 0.0001). No differences in *avtrv1a2* expression were observed between lineages in HW conditions but a higher *avtrv1a2* expression level was measured in ASW compared to MSW (Figure 3B). *avtrv2b1* and *avtrv2a1* mRNA levels were not different between AHW and ASW but were significantly higher in MHW compared to MSW (Figure 3C,D). *itr1* mRNA levels were slightly but not statistically higher in AHW compared to ASW but significantly higher in MHW compared to MSW (Figure 3E) (salinity effect: *p* < 0.0001). There were no differences in expression for *avtrv2b2*, *avtrv2a2* and *itr2* and *avtr2c* comparing salinities and lineages (Figure S2).

### 2.5. avtr and itr mRNA Expression in the Posterior Intestine

**Figure 4:**
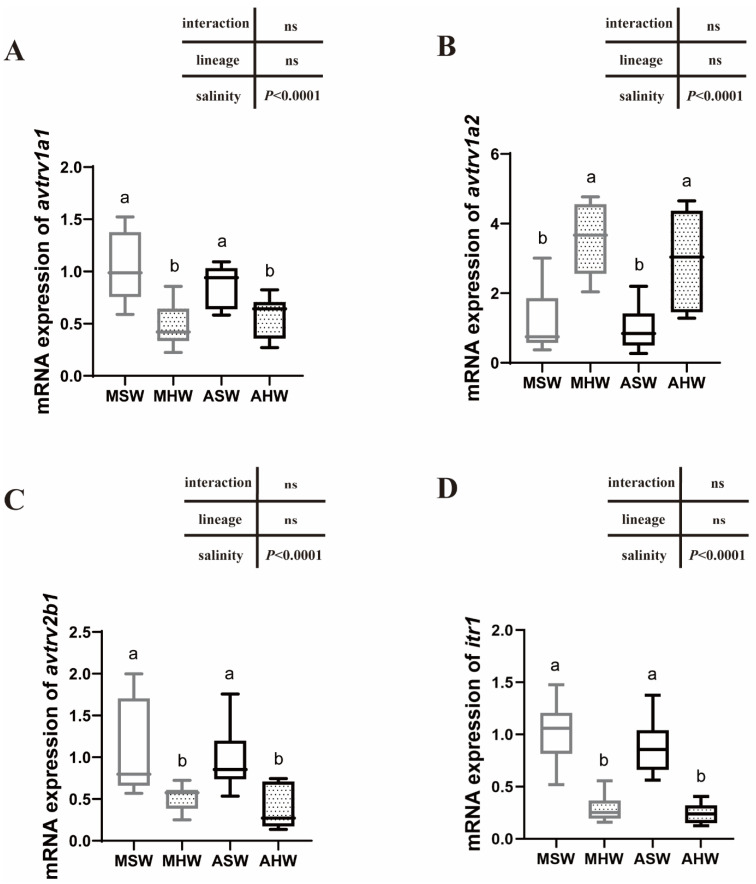
Relative *avtrv1a1* (**A**), *avtrv1a2* (**B**), *avtrv2b1* (**C**) and *itr1* (**D**) mRNA expression were measured in the posterior intestines of Mediterranean (M) and Atlantic (A) European sea bass maintained in seawater (SW) and hypersaline water (HW). Different letters denote significant differences between groups (two-way ANOVA followed by Tukey’s test, *p* < 0.05, *n* = 11). Data are represented as the median, first and third quartile (box), minimum and maximum values. ns: non significant.

In the posterior intestine, *avtrv1a2* was significantly higher in HW compared to SW of whatever lineages (*p* < 0.0001) (Figure 4B) and no differences between lineages were detected. For *avtrv1a1*, *avtrv2b1* and *itr1*, an inverse relation was observed with higher expression levels in SW than in HW whatever the lineages (*p* < 0.0001) (Figure 4A,C,D). There was no difference in expression between different conditions for *avtrv2a1*, *avtrv2a2*, *avtrv2b2*, *avtrv2c* and *itr2* (Figure S3).

### 2.6. Principal Component Analysis (PCA)

**Figure 5:**
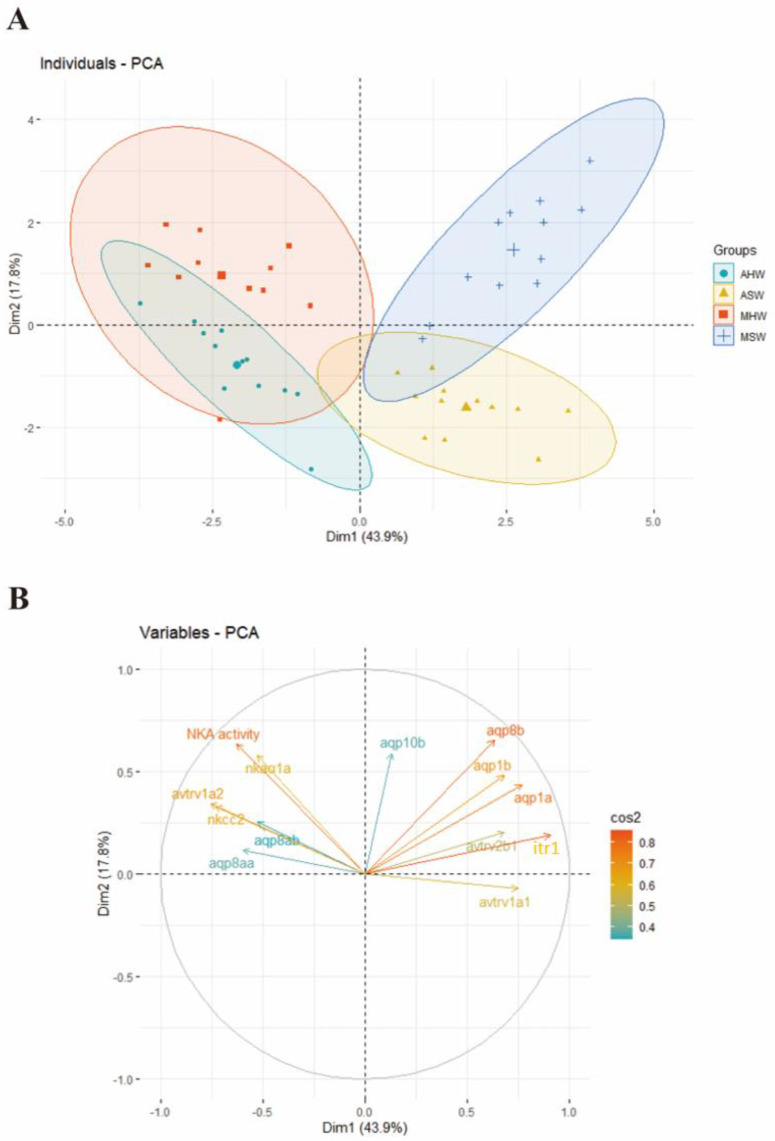
Loading plot of principal component analysis (PCA) representing the individuals (**A**) and measured variables (**B**) based on NKA activity, *nkaα1a*, *aqp8ab*, *aqp8aa*, *aqp8b*, *aqp10b*, *aqp1a*, *aqp1b*, *nkcc2*, *avtrv1a2*, *avtrv1a1*, *itr1* and *avtrv2b1* gene expressions in posterior intestines. Four conditions were compared (Mediterranean (M) and Atlantic (**A**) European sea bass maintained in seawater (SW) and hypersaline water (HW) in the (Dim1 × Dim2) coordination plane. Orange and green colors in (**B**) respectively represent strong and weak cos^2^ values. Ellipses in (**A**) group *D. labrax* from the four conditions (MSW in blue crosses, MHW in red squares, ASW in yellow triangles and AHW in green circles).

PCA results were obtained from the posterior intestine for gene expression levels of *avtr* and *itr* that showed different expression levels between conditions (*avtrv1a2*, *avtrv1a1*, *avtrv2b1*, *itr1*) as well as Na^+^/K^+^-ATPase (NKA) activity and expressions of genes involved in ion transport and water balance previously analyzed in the same individuals by Cao et al. [36] that showed different expression patterns between conditions (aquaporins: *aqp1a*, *aqp1b*, *aqp8b*, *aqp8ab*, *aqp8aa*, *aqp10b*, Na^+^/K^+^-ATPase α1a (*nkaα1a*) and Na^+^/K^+^/2Cl^−^ cotransporter 2 (*nkcc2*)) (Figure 5). Principal component 1 (PC1) described 43.9% of the original information whereas PC2 described 17.8%, making a cumulative percentage of 61.7%. PCA individual factor maps were shown in Figure 5A and showed a clear structuration along the PC1 axis with SW conditions on the right side of the biplot and hypersaline conditions on the left part of the biplot. A population structuration was observed along the PC2 axis but only in seawater conditions.

For the investigation of the contributors to the principal component, the variables in PC1 and PC2 were compared (Figure 5B). *avtrv1a2* grouped together with *aqp8aa*, *aqp8ab* and *nkcc2* with positive loading on the left upper side of the biplot. NKA activity and *nkaα1a* also grouped together on the left upper side. *avtrv1a1*, *avtrv2b1*, *itr1*, *aqp1a*, *aqp1b* and *aqp8b* appeared with a positive loading on the right side of the biplot.

## 3. Discussion

### 3.1. Diversity of European Sea Bass AVTR and ITR Receptors

Teleost species have a multitude of AVTR and ITR with generally seven or eight receptor family members including two ITR (ITR1, ITR2), two V1A (V1A1, V1A2), two V2A (V2A1, V2A2) and a different combination of one or two V2B (V2B1, V2B2) as well as one or no V2C (or V2B-like) [20,41]. Compared to mammals, teleost lack V1B sequences (Figure 1). Mammals have only one V1A, V1B, OTR, V2A and V2B. V2C is found in the chicken genome but not in human or mouse genomes [26] and its role in vertebrates still remains unknown. European sea bass harbor nine receptor family members with duplicated sequences of V2B (V2B1, V2B2) as well as the presence of V2C. The V2C receptor subtype has been previously reported as V2B-like or V2-like in zebrafish, three-spined stickleback and *D. labrax* (Tine et al., 2014, Ocampo et al., 2012) and has been renamed V2C by [26]. During evolution, V2C has been lost independently several times in the teleost lineages which explains the presence of V2C in a few species only [42]. Interestingly, V2C sequences in mammals share more similarities with fish V2B than with fish V2C (Figure 1). The presence of duplicate genes of V2B have been demonstrated in a few teleost species only as the pupfish (*Cyprinodon nevadensis amargosae*) [43], the brown trout (*S. trutta*) [42], Southern platyfish (*X. maculatus*) and Nile tilapia (*O. niloticus*) (Figure 1).

The diversification and interspecific difference regarding the number of AVTR genes in fish could be linked to the diversity of habitats in terms of salinity, various life cycles (migratory vs. sedentary) and different osmoregulatory strategies (for example in cartilaginous vs. actinopterygian species). A clear link between the number of gene copies and the degree of euryhalinity could not be established [41]. Further studies on the evolution and function of each specific paralog are therefore necessary to unravel their role in fish adaptive and acclimatory strategies.

Amino acid alignments of V1A1 and V1A2 (Figure S1) clearly showed a high conservation of putative AVT ligand binding sites previously identified in different studies [44], which assumes common AVT/IT binding sites between the two paralogs. A high conservation of amino acids involved in the oxytocin ligand selectivity, binding and signaling was observed within ITR and also AVTR sequences. Y209 present in *D. labrax* in ECL2, was only observed in ITR/OTR sequences and not in AVTR and is known in humans to interact with the oxytocin hormone [38].

### 3.2. V1A-Type Receptor and ITR1 Are Highly Expressed in the Intestine

A multitude of AVTR paralogs are observed in fish, which raises the question of the function of each paralog in the intestinal epithelium. The control of transepithelial ion uptake and solute-coupled water uptake are essential intestinal functions in marine teleost species that contribute to avoiding dehydration [10]. AVTR and ITR paralogs are particularly interesting to address in *D. labrax* due to the multitude of gene paralogs that exist in this species compared to others. We showed that the two *v1a* genes are the most expressed paralogs in Mediterranean sea bass maintained in their native salinity. *v1a2* was around three times more expressed than *v1a1* in the anterior and posterior intestines. The high expression of *v1a2* in fish intestine and notably the rectum has been previously reported in the gilthead sea bream *S. aurata* by [11,15]; however, contrary to those studies, *D. labrax v1a2* did not show a higher expression in distal parts of the intestine compared to anterior parts [15]. These authors have also shown that *v2*-type receptor expression is high and even higher than *v1a* in the anterior intestine. However, no distinction has been made between *v2*-type receptor paralogs, and the primers used in qPCR analyses might not distinguish between *v2*-type receptor subtypes. In European sea bass, where specific primers have been developed for each paralog, *v2*-type receptors are less expressed than *v1*-type receptors whatever the considered paralog. *v1*-type receptors are therefore considered as main AVTR receptors in European sea bass intestine. Among ITR receptors, *itr1* is the main paralog in both intestinal regions with a 50 to 80 higher expression than *itr2* in the anterior and posterior intestines. *itr1* was also more expressed than *itr2* in the intestines of ricefield eels [45].

### 3.3. Expression of AVTR Following Hypersalinity Transfer

*D. labrax* arginine vasotocin and isotocin receptor paralogs were examined in both intestinal regions following a transfer to hypersalinity. *v1a2*-type receptor showed a two- to four-fold upregulation in the European sea bass intestine after transfer to hypersaline water at 55 ‰, suggesting that *v1a2* is the most important AVTR paralog to mediate AVT responses to long-term hypersalinity challenge. An increased *v1a2* expression was also shown in a time-course study using the whole intestines of marbled eel *A. marmorata*, following a transfer of eels from fresh water to brackish and seawater [27].

Hypersalinity might induce a stress response leading to increased *v1a2* expression that could be triggered by stress hormones such as cortisol. Gilthead sea bream *S. aurata* implanted with cortisol showed an increased *v1a2* expression in gills and head kidneys, however, the intestine has not been analyzed so far [46].

Martos-Sitcha et al. [15] showed that a two-month acclimation from 35 to 55% showed a different trend in sea bream *v1a2* expression patterns in the rectum with lower expression at 55 compared to 35%. In *S. aurata* anterior intestines, no change in *v1a2* expression was observed between these two salinities. However, *v1a2* transcripts were overexpressed at the gill level following a hyper- and hypo-osmotic challenge [11,15]. An overexpression at high salinity (55‰) was also observed in the gills of the euryhaline Amargosa pupfish (*C. nevadensis amargosae*), but only following a short-term challenge (1 day) [16]. Overall, these studies indicate that a salinity challenge acts as a trigger for *v1a2* receptor overexpression in fish osmoregulatory tissues with, however, tissue-specific differences as well as differences in the timing of transcript abundance changes following high salinity challenge.

It is common to find two copies of *v1a* in teleost fish that arose from duplications in the early teleost 3R WGD (Whole Genome Duplication) event [42]. In the sea bass intestine, we clearly showed that *v1a1* and *v1a2* are differently regulated following hypersalinity stress and their function has thus diverged, *v1a2* being involved in the response to hypersalinity whereas *v1a1* is rather downregulated at salinities higher than seawater. A recent study in blue tilapia (*O. aureus*) has also compared anterior intestine AVTR expression following salinity increase from fresh water to 22% and showed a 4.5-fold increase in *v1a2* expression and no change for *v1a1* and other AVTR and ITR paralogous genes [47]. This confirms the role of *v1a2* in the response to high salinity. More studies are required in different species to better understand the role of each paralog in osmoregulation, as the expression of V1A paralogs seems to be triggered by different environmental factors.

Among the other AVTR/ITR paralogs investigated in *D. labrax*, hypersalinity triggered an increased *v2b1* and *itr1* expression in the anterior intestine and had an inverse effect on the posterior intestine with a significantly lower expression at 55%. The similarity in the expression patterns of both of these genes is intriguing and is also clearly shown in the PCA plot representing measured variables in the posterior intestine where *v2b1* and *itr1* group together. This suggests that the expression of both of these genes is controlled by a common factor or they might function together.

### 3.4. Functional Link between AVTR/ITR and Solute-Couped Water Uptake

PCA analysis showed that there was a strong correlation between *avtrv1a2* and the main ion transporters (NKA activity and expression of *nka α1a*, *nkcc2*) involved in ion uptake in the posterior intestine. We have previously shown that the expression of *nka α1a*, *nkcc2* and NKA activity was high in Mediterranean and Atlantic sea bass challenged to HSW [36]. The aquaporins, *aqp8ab* and *aqp8aa*, were also correlated with *avtrv1a2* and main ion transporters. Previous studies have shown that NKCC2 and AQP8ab were apically located in sea bass enterocytes, and AQP8 protein and gene expression increased upon hypersalinity transfer [36]. Altogether, *avtrv1a2* seems to be a valid candidate for regulating a coordinated response that would lead to an increased solute-coupled water uptake upon hypersalinity. For further studies, the localization of AVTRV1A2 in intestinal tissues as well as functional studies would be useful for confirming the potential functional link between AVTRV1A2 and solute-coupled water uptake in sea bass.

As mentioned above, *v2b1* and *itr1* showed a strong correlation and seem to have similar expression patterns to *aqp1a*, *aqp1b* and *aqp8b*. These three aquaporins are less expressed in HSW than in SW with, however, some differences among sea bass lineages [36]. In sea bream, IT has been suggested to regulate aquaporin 1 function and water absorption within the intestine [10,28]. Our results show similar expression patterns between *itr1* and *aqp1* paralogs, suggesting a functional link between ITR1 and AQP1a and 1b following hypersaline stress.

### 3.5. Intraspecific Differences in the Expression Patterns of Arginine Vasotocin and Isotocin Receptors

Salinity influences phenotypic traits in aquatic organisms and can be a driver for local adaptation when organisms evolve in different environments [48,49,50,51]. Genetic differences between Atlantic and Mediterranean lineages could be responsible for different physiological responses triggered by salinity and other abiotic factors [52,53,54]. Intraspecific differences in salinity tolerance have only been recently investigated in sea bass of Mediterranean and Atlantic origins [36,37]. To our knowledge, no study has addressed intraspecific difference in endocrine control. Comparing the responses of *D. labrax* from different origins (Mediterranean vs. Atlantic) at posterior intestine level, we clearly showed that the main differences in physiological traits occurred in close-to-native salinities, in seawater, and were due mainly to ion transporters and aquaporin expression patterns but not to AVTR and ITR, which showed similar expression patterns between lineages.

More generally, intraspecific differences in gene expression traits related to osmoregulation mainly exist in native salinities and not during hypersaline challenge whatever the intestinal section investigated. For example, MSW showed higher *avtrv1a1* and lower *avtrv1a2* expression levels than ASW in anterior intestines. As discussed before, *avtrv1a2* seems important for solute-coupled water uptake in posterior intestines. From previous studies, we know that MSW have a higher blood osmolality than ASW as well as higher NKA activity in the anterior intestine [36,37], suggesting a higher need for water uptake. The exact role and trigger of specific AVTR remains an open question in fish.

## 4. Materials and Methods

### 4.1. Phylogenetic Analysis of AVT and IT Receptors and Amino Acid Analysis

Amino acid sequences of *D. labrax* AVT V1A-type receptors (V1A1 and V1A2), the V2-type receptors V2A1, V2B1, V2B2 and V2C as well as the ITR1 and ITR2 (Table S1) were identified in the genome of *D. labrax* (http://seabass.mpipz.mpg.de/cgi-bin/hgGateway) (accessed on 24 June 2012) and were added to the dataset of Lagman et al. (2013) [26] (http://dx.doi.org/10.6084/m9.figshare.707336) (accessed on 24 June 2013). Multiple amino acid alignments were performed using MAFFT [55] and ambiguous regions were removed with Gblocks V0.91b [56]. The best model of evolution was selected using Modelgenerator V.85 [57] following the corrected Akaike Information Criterion (with four discrete gamma categories) and was used to construct a phylogenetic tree. Bayesian posterior probabilities were computed with MrBayes 3.2.1 [58]. Two different runs with four incrementally heated simultaneous Monte Carlo Markov chains were conducted over one million generations. Trees were sampled every 100 generations to produce 10,000 trees. In order to estimate posterior probabilities, 25% of the trees were discarded as a burn-in stage, observing when the average standard deviation of split frequency (ASDSF) values dropped below 0.01. The tree was rooted with the common octopus OTR, CTR1 and CTR2 sequences.

Multiple amino-acid alignments were performed using the Geneious alignment tools and functional domain regions were identified based on Mayasich and Clarke, (2020) [44]. Sequence logos were created for the functional domain regions in AVT V1A-type receptors (V1A1 and V1A2) and ITR1 using WebLogo Version 3 (https://weblogo.threeplusone.com/). Amino acid sequences from mammalian and fish species were used (Table S1).

### 4.2. Experimental Design

European sea bass *D. labrax* were collected from West Mediterranean and Atlantic lineages maintained in seawater (SW: 36%) at the Ifremer Station (Palavas-les-Flots, Hérault, France). Sea bass from both lineages were brought to Montpellier University and maintained for another week in 3500 L tanks at 20 °C and a constant photoperiod (12hL/12hD). The tanks contained natural seawater from the Mediterranean Sea slightly diluted with tap water to reach a salinity of 36%. Twelve fish per condition were transferred to smaller 200 L tanks containing either hypersaline water (HW: 55%) or seawater (SW: 36%). Hypersaline water was made by adding sea salt (Instant Ocean, Blacksburg, USA) to SW. Fish were maintained at this salinity for two weeks until sampling. In this study, four conditions were compared: West Mediterranean *D. labrax* maintained at 36% (MSW) and 55% (MHW) as well as North Atlantic *D. labrax* maintained at 36% (ASW) and 55% (AHW). Water was mechanically and biologically filtered (Eheim System, Lens, Pas-de-Calais, France). Oxygen levels, nitrogen levels, temperature and salinity were regularly checked. A siphon tube was used daily to remove 10% of the water and replace it with clean water at the same salinity and temperature. Fish were fed daily ad libitum with granules (Aphymar, Mèze, Hérault, France) until two days before sampling. Fish were anesthetized using benzocaine at 50 ppm prior to any manipulation. Fish mean fork length and body weight were determined before sampling. Eleven fish per condition were analyzed with qPCR. The intestine was equally subdivided into three segments, a proximal (corresponding to the first segment) and a distal segment (corresponding to the last segment) that will be called anterior and posterior intestines. For mRNA analysis, samples were flash frozen in liquid nitrogen and stored at −80 °C until analysis. The animal experimentations followed the rules of the European Union (Instruction 86/609) and French law (decree 87/848).

### 4.3. RNA Extraction and Complementary cDNA Synthesis

The Nucleospin^®^ RNA (MACHEREY NAGEL GMbH Co.KG, Düren, Germany) extraction kit was used for total RNA extraction and DNAse treatment. The A260/A280 ratio was determined using a NanoDrop^®^ One Spectrophotometer (ThermoFisher, Waltham, MA, USA). First-strand cDNA synthesis was performed with 1 µg RNA using qScript™ cDNA SuperMix (Quanta Biosciences™, Beverly, MA, USA) following the manufacturer’s instructions.

### 4.4. Quantitative Real-Time PCR (qPCR)

The primers used in this study are indicated in Table 1. 0.75 μL of SensiFAST™ SYBR^®^ No-ROX Kit (Bioline, London, UK), 0.037 μL of each primer (at 0.4 μM), 0.21 μL of ultra-pure water and 0.5 μL of diluted cDNA were dispensed into a 384-well reaction plate in an Echo^®^525 liquid handling system (Labcyte Inc., San Jose, CA, USA). Generated standard curves helped to determine the optimal cDNA dilution for each primer pair that was set as 1/16. Each sample was run in duplicate. The qPCR procedure consisted of an initial denaturation (1 cycle, 95 °C, 150 s) followed by 45 cycles of denaturation (95 °C, 15 s), hybridization (60 °C, 5 s), elongation (72 °C, 10 s) and a final step (1 cycle, 40 °C, 30 s). The amplification specificity was controlled by a melting curve program. The no-template control used ultra-pure water in this program. Efficiencies were measured and are indicated in Table 1. Elongation factor (*ef1α*), ribosomal protein L13 (*l13*) and ribosomal protein S30 fusion gene (*fau*) were used as reference genes. The geometric mean of the three reference genes normalized the expression levels using the ∆∆Ct calculation method and the efficiency of each primer pair. The MSW condition was used as a control condition when comparing gene expression levels at different salinities and lineages. A reference Ct value corresponding to the Ct average of analyzed genes in all individuals was used when comparing gene expression levels in the MSW condition. Relative quantifications were performed using the method of Vandesompele et al. (2002) [59]. The threshold cycle (Ct) of the three reference genes did not change at the tested conditions (*p* < 0.05).

### 4.5. Statistical Analyses

Statistical analyses were performed using GraphPad Prism (version 8, GraphPad Software Incorporated, La Jolla, CA, USA). Normality and homogeneity tests were verified using the D’Agostino–Pearson and Bartlett tests. If the data fit with these conditions, a two-way ANOVA analysis was performed with salinity and the lineage or the tissue and the gene as main factors followed by a Tukey’s multiple comparisons test (Table 2). Data are represented as box and whisker plots (from the first quartile to the third quartile) showing median, minimum and maximum values. Principal component analysis (PCA) was performed using standardized individual gene expression values and NKA activities using Rstudio (version 1.2.5001) and the factoextra, missMDA and FactoMineR packages [60].

## 5. Conclusions

To conclude, this study provides evidence of the presence of seven *avtr* and two *itr* in European sea bass. For the first time, transcript responses for all teleost arginine vasotocin and isotocin receptors were examined in the anterior and posterior intestines in seawater and during hypersalinity acclimation. *avtrv1a1* and *avtrv1a2* are salinity responsive genes, however, with opposite expression patterns after the transfer of fish to hypersalinity. A major role of the *avtrv1a2* receptor is attributed to solute-coupled water uptake in the posterior intestine.

## Figures and Tables

**Table 1:** Primer sequences used for qPCR in this study. F: forward primer; R: reverse primer. Sequences ID indicate gene sequences from the sea bass genome or Genbank identification numbers when available.

Sequences ID	Target Gene	Primer Name	Sequence (from 5′ to 3′)	Amplicon Size	Efficiency
DLAgn_00209950	*avtrv1a1*	*avtrv1a1*-F *avtrv1a1*-R	CGCGCCAAATTACGCACG TAGCGGGCGGCAGCACG	259	1.858
DLAgn_00170900	*avtrv1a2*	*avtrv1a2*-F *avtrv1a2*-R	CAGTCTCTGGTTTTTAGTCCCAC AAGGTTGTGTTGTACATTGCCA	194	2.025
DLAgn_00207720	*avtrv2c*	*avtrv2c*-F *avtrv2c*-R	AGAGCTGGGATTTTGGGTTTG CAAGTATTTTACGGACCGGC	209	2.062
DLAgn_00125320	*avtrv2a2*	*avtrv2a2*-F	CAACAACCCTCTGCACCAG	234	2.22
		*avtrv2a2*-R	TGCGCCAGCAGAGGGCAG		
DLAgn_00092800	*avtrv2b1*	*avtrv2b1*-F	TCAGTAAAGACTCTGTGAGCAGC	174	2.001
		*avtrv2b1*-R	CTGTCTGTAAAGGCAAAAGTGCT		
DLAgn_00089180	*avtrv2b2*	*avtrv2b2*-F	TGCGTCTCTCTCATCTTCGG	165	2.083
		*avtrv2b2*-R	CACCGTGAGAATGGGCAGG		
DLAgn_00131190	*avtrv2a1*	*avtrv2a1*-F	GCACGCCTAATGTACAAAGCAG	213	2.03
		*avtrv2a1*-R	CTCCTGCAGCAGTAACATCCAT		
DLAgn_00093990	*itr2*	*itr2*-F	CTGCCGACAGTACCTACCCT	145	2.188
		*itr2*-R	TCCTGGCCCTCCATTGCT		
DLAgn_00093190	*itr1*	*itr1*-F	AACCTCCAAAGGCAACACGC	214	1.869
		*itr1*-R	CATGGAGATGATGAAGGGCA		
FM004681	*Fau*	*fau*-F *fau*-R	GACACCCAAGGTTGACAAGCAG GGCATTGAAGCACTTAGGAGTTG	150	1.851
AJ866727	*ef1α*	*ef1α*-F *ef1α*-R	GGCTGGTATCTCTAAGAACG CCTCCAGCATGTTGTCTCC	239	1.853
DLAgn_00023060	*l13*	*l13*-F *l13*-R	TCTGGAGGACTGTCAGGGGCATGC AGACGCACAATCTTGAGAGCAG	148	1.873

**Table 2:** Two-way ANOVA results of data with salinity and lineage as the main factors. ns: not significant, * *p* < 0.05, ** *p* < 0.01, **** *p* < 0.0001. N = 11 per condition.

	Interaction	Salinity	Lineage
**mRNA expression**			
*AI-avtrv1a1*	**	ns	****
*AI-avtrv1a2*	*	****	ns
*AI-avtrv2a1*	ns	*	ns
*AI-avtrv2a2*	ns	ns	ns
*AI-avtrv2b1*	****	****	**
*AI-avtrv2b2*	ns	ns	ns
*AI-avtrv2c*	ns	ns	ns
*AI-itr2*	ns	ns	ns
*AI-itr1*	ns	****	*
*PI-avtrv1a1*	ns	****	ns
*PI-avtrv1a2*	ns	****	ns
*PI-avtrv2a1*	ns	ns	*
*PI-avtrv2a2*	ns	ns	ns
*PI-avtrv2b1*	ns	****	ns
*PI-avtrv2b2*	ns	ns	ns
*PI-avtrv2c*	ns	ns	ns
*PI-itr2*	ns	ns	ns
*PI-itr1*	ns	****	ns

## Data Availability

The data are available upon request to the corresponding author.

## Supplementary Materials

The supporting information can be downloaded at: https://www.mdpi.com/article/10.3390/ijms232113636/s1.
