# Supplementary material for: Hypo-Osmoregulatory Roles of Vasotocinergic and Isotocinergic Systems in the Intestines of Two European Sea Bass Lineages
Source: Int J Mol Sci. 2022 Nov 7;23(21):13636. doi: 10.3390/ijms232113636 (PMC9655083; doi:10.3390/ijms232113636)

Table S1: AVTRV and ITR sequences used in phylogenetic analyses and sequences

Fish species	Genes	Sequence ID	Fish species	Genes	Sequence ID
Dicentrarchus labrax	V2B1	*DLAgn_00092800*	***Lepisosteus oculatus***	ITR	*ENSDARP00000050113_1*
	V2B2	*DLAgn_00089180*		V2B	*ENSLACP00000015306_1*
	V2A1	*DLAgn_00131190*		V1A	*ENSGACP00000004706_1*
	V2A2	*DLAgn_00125320*		V2A	*ENSGACP00000014075_1*
	V1A1	*DLAgn_00209950*		V2C	*JH591411.1*
	V1A2	*DLAgn_00170900*	***Gallus gallus***	V2C	*ENSGALG00000009497*
	V2C	*DLAgn_00207720*		V1A	*ENSGALG00000009808*
	ITR1	*DLAgn_00093190*		OTR	*ENSGALG00000003138*
	ITR2	*DLAgn_00093990*		V1B	*ENSGALG00000000788*
Oreochromis niloticus	V1Aa	*XM_003443925.5*		V2C	*ENSMGAG00000009257*
	V2A1	*XM_003448162.5*	***Danio rerio***	V2C	*ENSDARG00000076690*
	V2B2	*XM_003442789.5*		ITR1	*ENSDARG00000033956*
	V2B1	*XM_025907435.1*		V2B	*ENSDARG00000076797*
	ITR1	*XM_003441444.5*		ITR2	*ENSDARG00000044175*
	ITR2	*XM_003448399.3*		V2A1	*ENSDARG00000029219*
Gasterosteus aculeatus	V2C	*ENSGACG00000018884*		V2A2	*ENSDARG00000007436*
	V2A1	*ENSGACG00000008681*		V1Aa	*ENSDARG00000077083*
	V2A2	*ENSGACG00000010636*	***Latimeria chalumnae***	ITR	*ENSLACG00000018362*
	V1Aa	*ENSGACG00000003589*		V1B	*ENSLACG00000013680*
	V1Ab	*ENSGACG00000000737*		V1A	*ENSLACG00000014523*
	V2B	*ENSGACG00000000754*		V2A	*ENSLACG00000014898*
	ITR1	*ENSGACG00000000914*		V2B	*ENSLACG00000013474*
Xiphophorus maculatus	V1Aa	*ENSXMAG00000017820*	***Oryzias latipes***	ITR1	*ENSORLG00000000719*
	ITR2	*ENSXMAG00000016299*		V2B	*ENSORLG00000001494*
	ITR1	*ENSXMAG00000001524*		ITR2	*ENSORLG00000003297*
	V2A1	*ENSXMAG00000010783*		V1Aa	*ENSORLG00000002126*
	V2A2	*ENSXMAG00000009554*		V2A1	*ENSORLG00000007088*
	V2C	*ENSXMAG00000009218*		V2A2	*ENSORLG00000016671*
	V2B(1)	*ENSXMAG00000001906*		V1Ab	*ENSORLG00000017245*
	V2B(2)	*ENSXMAG00000002230*	***Takifugu rubripes***	V2B	*ENSTRUG00000001010*
Anolis carolinensis	V2A	*ENSACAG00000021016*		V2A2	*ENSTRUG00000000643*
	V2C	*ENSACAG00000000005*		V2A1	*ENSTRUG00000015164*
	V1A	*ENSACAG00000000443*		V1Ab	*ENSTRUG00000016314*
	V1B	*ENSACAG00000012849*		V1Aa	*ENSTRUG00000006025*

Fish species	Genes	Sequence ID	Fish species	Genes	Sequence ID
Anolis carolinensis	OTR	*ENSACAG00000011241*	***Takifugu rubripes***	ITR2	*SINFRUG00000140687*
Xenopus tropicalis	OTR	*ENSXETG00000001441*		ITR1	*ENSTRUG00000003223*
	V2A	*ENSXETG00000014233*	***Mus musculus***	OTR	*ENSMUSG00000049112*
	V2C	*ENSXETG00000015741*		V1A	*ENSMUSG00000020123*
	V1A	*ENSXETG00000018781*		V2A	*ENSMUSG00000031390*
	V1B	*ENSXETG00000005930*		V1B	*ENSMUSG00000026432*
Homo sapiens	V1B	*ENSG00000198049*	***Ciona intestinalis***	VPR	*ENSCING00000006620*
	OTR	*ENSG00000180914*	***Octopus vulgaris***	OPR	*AB158496.1*
	V1A	*ENSG00000166148*		CTR1	*AB158493.1*
	V2A	*ENSG00000126895*		CTR2	*AB158495.1*

Fig. S1 Sequence logos in selected regions of mammalian and fish V1a/V1a1 compared to *D. labrax* V1a1 (first two lines), mammalian and fish V1a/V1a2 compared to *D. labrax* V1a2 (third and fourth line) and mammalian and fish OTR/ITR1 compared to *D. labrax* ITR1. Black asterisks indicate putative OXT/AVP ligand binding amino acids in mammalian receptors (Chini and Fanelli, 2000; Koehbach et al., 2013). Red asterisks indicate important residues for AVT binding (Acharjee et al., 2004; Chini and Fanelli, 2000). Red boxes show important residues for oxytocin ligand selectivity, binding and signaling (Koehbach et al., 2013). Green boxes show amino acid differences between *D. labrax* V1a1 and V1a2. Sequence numbering corresponds to the human OXTR sequence (Accession no. NP_001341583.1).

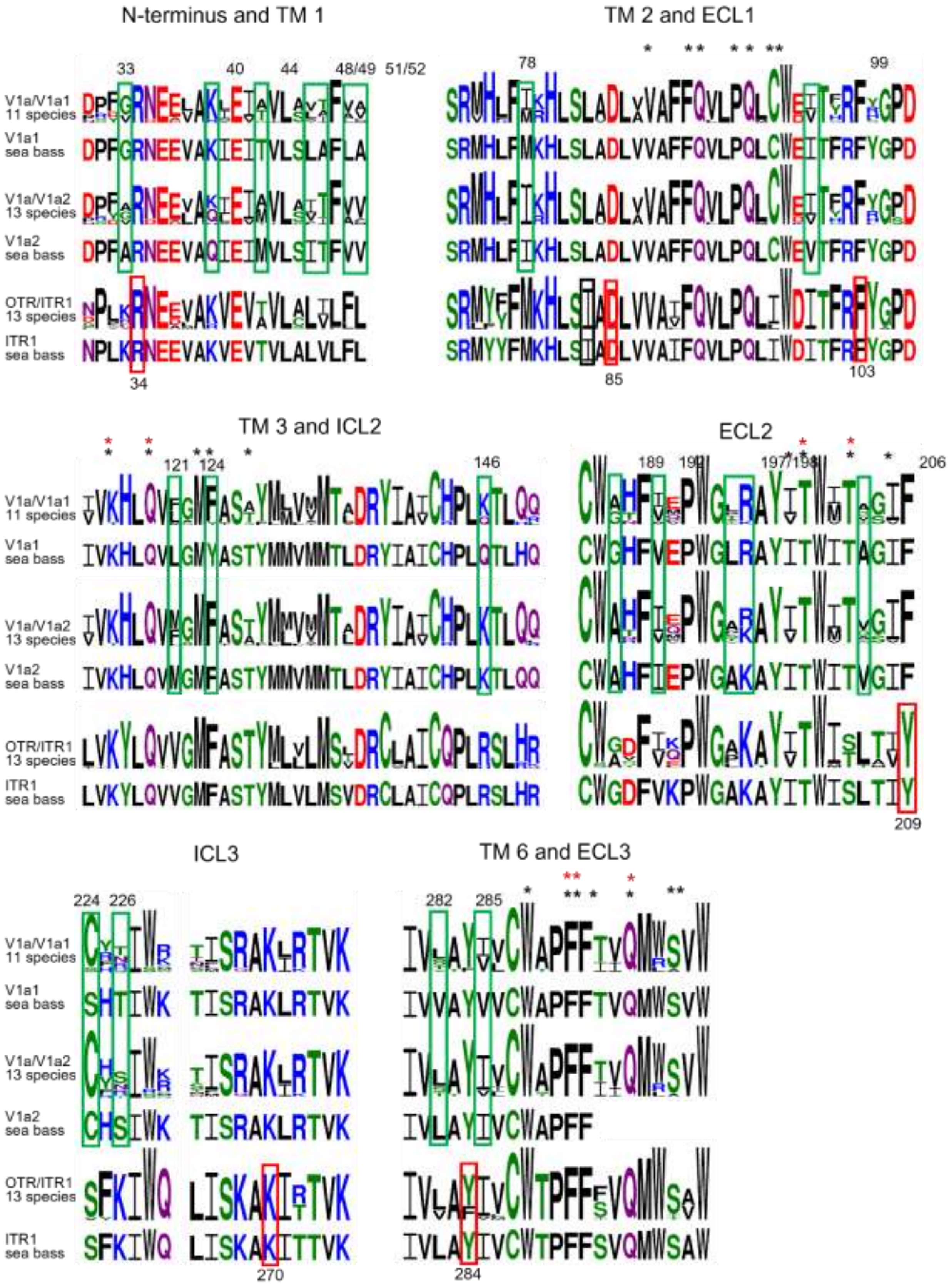
N-terminus and TM 1
TM 2 and ECL1
TM 3 and ICL2
ECL2
ICL3
TM 6 and ECL3
V1a/V1a1 11 species
V1a1 sea bass
V1a/V1a2 13 species
V1a2 sea bass
OTR/ITR1 13 species
ITR1 sea bass
33
40
44
48/49
51/52
34
78
99
85
103
121
124
146
189
197/198
206
209
224
226
270
282
285
284

Fig. S2 Relative *avtrv2b2*, *avtrv2a2*, *avtrvc* and *itr2* mRNA expression were measured in the anterior intestines of Mediterranean (M) and Atlantic (A) European sea bass maintained in seawater (SW) and hypersaline water (HW). Different letters denote significant differences between groups (two-way ANOVA followed by Tukey's test, $P < 0.05$, N = 11). Data are represented as the median, first and third quartile (box), minimum and maximum values.

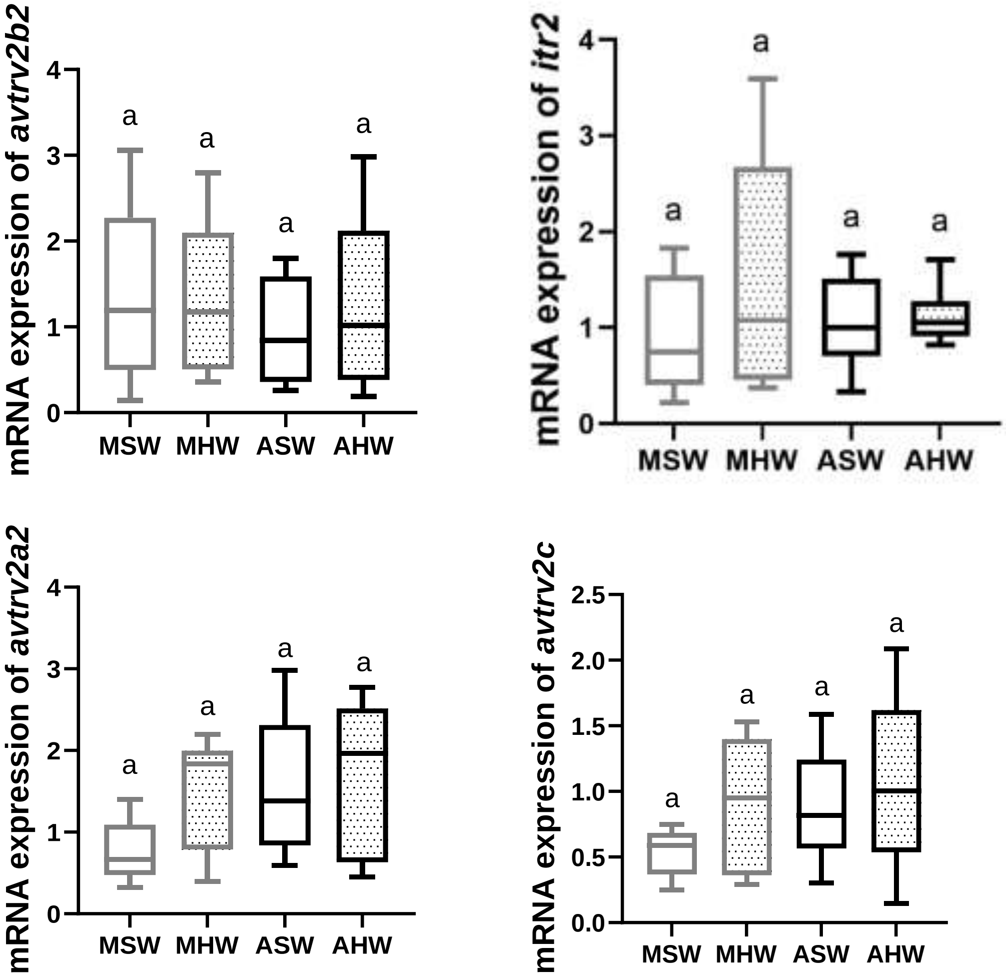

Fig. S3 Relative *avtrv2a1*, *avtrv2a2*, *avtrv2b2*, *avtrv2c* and *itr2* mRNA expression were measured in the posterior intestines of Mediterranean (M) and Atlantic (A) European sea bass maintained in seawater (SW) and hypersaline water (HW). Different letters denote significant differences between groups (two-way ANOVA followed by Tukey's test, $P < 0.05$, N = 11). Data are represented as the median, first and third quartile (box), minimum and maximum values.

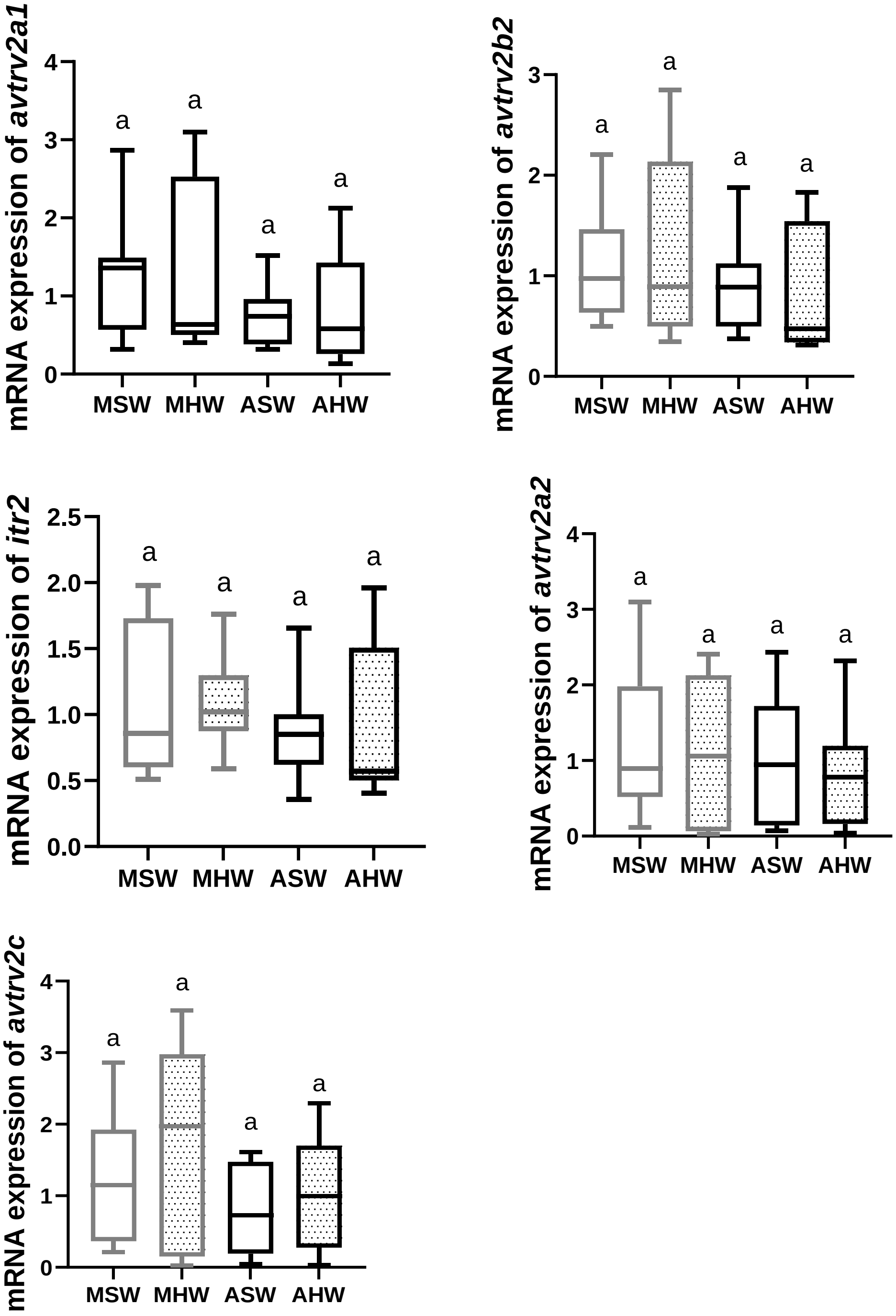

Supplement: Supplementary file 1 [file ijms-23-13636-s001.zip › ijms-1981023-supplementary.pdf]
